# Impact of von Willebrand Disease on Women's Health Outcomes: A Matched Cohort Database Study

**DOI:** 10.1089/jwh.2022.0082

**Published:** 2022-09-15

**Authors:** Katrina Wilcox Hagberg, Susan Jick, Ping Du, Françoise Truong Berthoz, Gülden Özen, Spiros Tzivelekis

**Affiliations:** ^1^ Boston Collaborative Drug Surveillance Program, Lexington, Massachusetts, USA.; ^2^ Boston University School of Public Health, Boston, Massachusetts, USA.; ^3^ Takeda Development Center Americas, Inc., Cambridge, Massachusetts, USA.; ^4^ Takeda Pharmaceuticals International AG, Zürich, Switzerland.

**Keywords:** von Willebrand disease, women's health, heavy menstrual bleeding, hysterectomy

## Abstract

**Objective:**

To understand the impact of von Willebrand disease (VWD) on women's health, a retrospective cohort study was conducted using UK Clinical Practice Research Datalink (CPRD) GOLD database and Hospital Episode Statistics (HES) Admitted Patient Care data from 1988 to 2016.

**Materials and Methods:**

Hysterectomy and heavy menstrual bleeding (HMB) events were identified by recorded disease/clinical codes and compared in women with and without VWD (matched 1:10 by birth and CPRD record start years [±2 years], and general practice attended). Incidence rates and incidence rate ratios (IRR) were calculated; risks were estimated using the Kaplan–Meier method.

**Results:**

HMB was recorded after cohort entry in 388 of 1,335 women (29.1%) with VWD and 1,524 of 12,463 women (12.2%) without VWD. The cumulative incidence of HMB was higher in women with versus without VWD across all ages (*p* < 0.0001), and irrespective of prior HMB status (*p* < 0.001). Women with VWD were more likely to have HMB compared with women without VWD; IRR adjusted for age and prior HMB status was 2.74 (95% confidence interval [CI]: 2.44–3.07). Hysterectomy was recorded in 88 of 1,374 women (6.4%) with VWD and 320 of 12,791 women (2.5%) without VWD. The cumulative incidence of hysterectomy was higher for women with versus without VWD (*p* < 0.0001), and highest among women aged ≥30 years at cohort entry. Women with VWD aged 30 − 39 years were more likely to undergo hysterectomy than women without VWD; IRR adjusted for prior HMB was 3.58 (95% CI: 2.36 − 5.44).

**Conclusions:**

These findings highlight the substantial impact of VWD on women's health.

## Introduction

Von Willebrand disease (VWD) is an inherited bleeding disorder characterized by deficiency in the blood clotting protein von Willebrand factor (VWF), which mediates platelet functions and stabilizes coagulation factor VIII (FVIII) in the circulation.^1–3^ The overall global prevalence of VWD is estimated at 600 − 1,300/100,000 or 10/100,000 for symptomatic VWD requiring treatment.^4^ Patients with VWD present with a wide range of heterogeneous bleeding phenotypes, including easy bruising, prolonged bleeding from wounds or surgeries, joint bleeding, mucocutaneous bleeding, heavy menstrual bleeding (HMB), and potentially life-threatening bleeding involving the central nervous system and gastrointestinal tract.^1,5,6^

HMB or menorrhagia is often the primary symptom in women with VWD and can negatively impact their lives and short- and long-term health outcomes.^6–8^ HMB is often associated with symptomatic iron deficiency anemia, psychological stress, and reduced quality of life.^8^ It can impact work, school, and daily activities and can result in increased health care costs.^8^ Furthermore, women of reproductive age with VWD may need to be treated surgically with a hysterectomy because of HMB.^7^

The management of HMB has recently been reviewed by a multidisciplinary panel as part of the development of the international guidelines for the management of VWD published in 2021.^5^ However, there is still limited evidence to understand the burden and health impact of HMB in women with VWD. In addition, previous research has mainly focused on women with moderate or severe VWD seeking diagnosis or care at specialist centers,^7,9,10^ while limited data exist relating to unmet needs for women with VWD managed in the general practice setting. This retrospective cohort study used the United Kingdom (UK) Clinical Practice Research Datalink (CPRD) GOLD database and Hospital Episode Statistics (HES) Admitted Patient Care, Outpatient, and Accident and Emergency database to establish a better understanding of the potentially unmet needs of women with this bleeding disorder.

## Materials and Methods

### Data source

All available data in the UK CPRD GOLD database (January 1, 1988 through June 30, 2016) and HES database (January 1, 1997 through June 30, 2016) were used in this analysis.

CPRD GOLD is a large, prospectively collected, anonymized electronic medical record database encompassing over 500 UK National Health Service (NHS) general practices covering over 10 million patients and containing over 45 million person-years of follow-up. This is a population-based resource broadly representative in terms of age, sex, and minority distribution of the UK population.^11,12^ Because UK health care is universal and free, selection bias related to differential access to health care is minimized. CPRD GOLD contains demographic information, prescription details, clinical events, referrals, hospital admissions, health care resource use, and lifestyle details (*e.g.*, smoking, alcohol consumption). Details from inpatient and outpatient medical encounters are reported to patients' general practitioners (GPs) in the form of consultant and discharge letters, and GPs may code the information in the patient's electronic data. All patients in this study were from the general practice setting and therefore are representative of patients attending general practice care.

The HES Admitted Patient Care database contains deidentified/anonymized details of all admissions to NHS hospitals in England (a subset of the CPRD GOLD practices, with ∼60% of patients included in both databases). HES data comprise details of NHS hospital stays, including dates of admission and discharge, the primary diagnosis, additional diagnoses, and procedures; data are recorded using International Classification of Disease-10 codes (ICD-10).

### Study population and outcomes of interest

From CPRD GOLD, a cohort of women with VWD was selected on the basis of sex and a Read code (a clinical terminology system used in UK general practice) indicating a diagnosis of VWD (D304.00) recorded at any time in the patient's electronic record. Women who were assumed to have acquired von Willebrand syndrome because of a diagnosis of certain autoimmune disorders (*i.e.*, lymphoproliferative diseases, monoclonal gammopathies, systemic lupus erythematosus, hypothyroidism, essential thrombocythemia, polycythemia vera, myelofibrosis) or some cancers (*e.g.*, myeloproliferative neoplasms) were excluded (see Supplemental Appendix S1 for relevant Read codes). All other patients were assumed to have congenital VWD, regardless of age at first recorded VWD diagnosis. A cohort of women without VWD was also selected from the UK CPRD and matched (up to 10:1) to each woman with VWD based on birth year (±2 years), CPRD record start year (±2 years), and general practice attended (to match on HES data eligibility, geography, socioeconomic factors, and standard of medical care). Patients were categorized based on the timing of their first VWD diagnosis or match date and whether it occurred in the patient's “historical record” (before the patient's CPRD record start date) or in the patient's “active record” (after the patient's CPRD record start date).

To ensure inclusion of individuals at risk of developing each of the two study outcomes—HMB or hysterectomy—the cohort entry date was defined as either the date the patient turned 10 years of age or the patient's record start date, whichever was later. For the HMB analysis, women who had a hysterectomy before the cohort entry date or who were aged ≥65 years at cohort entry were excluded. Each woman was followed from cohort entry date to censoring date, which was defined as the date of the first recorded HMB event after cohort entry, hysterectomy date, date of becoming 65 years of age, date of CPRD record end, or death, whichever came first. The HMB index date was defined as the first HMB event recorded in CPRD GOLD or HES after cohort entry.

Hysterectomy analyses were restricted to female patients aged ≥10 years who did not have a record for hysterectomy before cohort entry. Each woman was followed from the cohort entry date to the censoring date, defined as hysterectomy date, date of CPRD record end, or death, whichever came first. The hysterectomy index date was defined as the first hysterectomy code recorded in the CPRD GOLD or HES data after cohort entry.

### Covariates

In addition to the matching criteria, differences in available data sources (CPRD GOLD data only or CPRD GOLD data plus eligibility for HES linkage) between the VWD cohort and the matched non-VWD cohort were assessed. The presence of the following covariates that are related to general health or gynecological risk factors at cohort entry (including events present in the historical record or that occurred before the age of 10 years) and at the HMB or hysterectomy date were also described in women with and without VWD: hypertension, diabetes, hypercholesterolemia, chronic kidney disease, liver disease, alcohol abuse, drug abuse, thyroid disorders, history of prior pregnancy, prior history of HMB, uterine fibroids, uterine polyps, endometriosis, pelvic infections, uterine prolapse, and uterine or cervical cancer. Age and HMB status were also used as time-varying covariates in the analyses.

### Statistical analysis

The demographics of the VWD and matched non-VWD cohorts were described. Patient-years were calculated from the cohort entry date through the censoring date, separately for each outcome. Byar's method was used to calculate the incidence rate (IR) with 95% confidence intervals (CI) of each outcome for women with or without VWD, both overall and stratified by age and HMB status. Poisson regression analysis was used to calculate incidence rate ratios (IRR) with 95% CIs of each outcome for women with VWD compared with women without VWD. The IRR for HMB and hysterectomy were adjusted for age and prior HMB.

The risk (cumulative incidence function) was also estimated for each outcome using the Kaplan–Meier method, both overall and stratified by age at cohort entry. Kaplan–Meier curves were compared using a log-rank test. For HMB, the cohorts were also stratified by prior history of HMB before cohort entry. Finally, Cox proportional hazard ratios (HRs) and their 95% CIs were estimated for each outcome in women with versus without VWD. Models that did not meet the proportionality assumption were identified by visual inspection and by use of the Schoenfeld residuals test. The HR and 95% CI for hysterectomy were estimated, adjusted for the competing risk of death using R package “cmprsk.” Demographic and IR analyses were performed using SAS 9.4 and all Kaplan–Meier estimates, Cox models, and competing risk analyses were performed using R version 4.0.2.

### Ethical review and copyright

This study is based, in part, on data from the CPRD obtained under license from the UK Medicines and Healthcare products Regulatory Agency. The data are provided by patients and collected by the UK NHS as part of their care and support. The interpretation and conclusions contained in this study are those of the authors alone. This study was approved by the Independent Scientific Advisory Committee (ISAC) for Medicines and Healthcare products Regulatory Agency database research, and the protocol was made available to the journal reviewers upon request (protocol No: 17_242R2A, Hospital Episode Statistics (HES) Copyright ^©^ [2018], reused with permission of The Health & Social Care Information Centre. All rights reserved).

Researchers can apply for a limited license to access CPRD data for public health research, subject to individual research protocols meeting CPRD data governance requirements. More details, including data specifications, license fees, and the application process, are available on the CPRD website (https://www.cprd.com).

## Results

### Heavy menstrual bleeding

In total, 1,335 women with VWD and 12,463 women without VWD were eligible for inclusion in the HMB analyses (Table 1; Supplementary Table S1). Most women with or without VWD (59.6% and 59.4%, respectively) had both CPRD GOLD data and eligibility for HES linkage. Most women (61.4% with VWD and 62.5% without VWD) were aged 10–29 years at cohort entry (median [range] 25 [10–64] years). Approximately 9.1% of women with VWD had a prior history of HMB recorded before cohort entry, compared with 1.9% of women without VWD.

**Table 1.** Characteristics of the Study Population Used for the Heavy Menstrual Bleeding Analysis: Women With or Without von Willebrand Disease Overall and Those Who had Heavy Menstrual Bleeding Diagnosed After Cohort Entry Date

	All patients in HMB analysis		Women with HMB diagnosed after cohort entry	
	Women with VWD (*n* = 1,335)	Women without VWD (*n* = 12,463)	Women with VWD (*n* = 388)	Women without VWD (*n* = 1,524)
Data source, *n* (%)	Cohort entry		HMB diagnosis	
CPRD data plus eligible for HES linkage	796 (59.6)	7,403 (59.4)	—	—
CPRD data only	539 (40.4)	5,060 (40.6)	267 (68.8)	1,128 (74.0)
HES data only	—	—	56 (14.4)	243 (15.9)
CPRD plus HES data	—	—	65 (16.8)	153 (10.0)
Year, *n* (%)	Cohort entry		HMB diagnosis	
1988 − 1999	533 (39.9)	4,981 (40.0)	85 (21.9)	313 (20.5)
2000 − 2009	614 (46.0)	5,767 (46.3)	195 (50.3)	728 (47.8)
2010 − 2016	188 (14.1)	1,715 (13.8)	108 (27.8)	483 (31.7)
Age, years, *n* (%)	Cohort entry		HMB diagnosis	
10 − 19	457 (34.2)	4,230 (33.9)	93 (24.0)	209 (13.7)
20 − 29	363 (27.2)	3,554 (28.5)	83 (21.4)	262 (17.2)
30 − 39	299 (22.4)	2,862 (23.0)	105 (27.1)	439 (28.8)
40 − 49	147 (11.0)	1,295 (10.4)	91 (23.5)	525 (34.5)
50 − 64	69 (5.2)	522 (4.2)	16 (4.1)	89 (5.8)
Mean (SD) age, years	26.3 (12.9)	25.9 (12.4)	30.5 (11.9)	34.8 (11.0)
Median (range) age, years	25 (10 – 64)	25 (10 – 64)	31.0 (10 – 55)	36.5 (10 – 59)
Covariates, *n* (%)	Before cohort entry		Before HMB diagnosis date	
HMB	122 (9.1)	237 (1.9)	NA	NA
Hypertension	25 (1.9)	160 (1.3)	16 (4.1)	73 (4.8)
Diabetes	8 (0.6)	75 (0.6)	NR	28 (1.8)
Hypercholesterolemia	NR	35 (0.3)	NR	17 (1.1)
Chronic kidney disease	0 (0.0)	6 (0.1)	NR	10 (0.7)
Liver disease	NR	9 (0.1)	0 (0.0)	NR
Alcohol abuse	23 (1.7)	139 (1.1)	17 (4.4)	51 (3.4)
Drug abuse	9 (0.7)	69 (0.6)	NR	19 (1.3)
Thyroid disorder	9 (0.7)	144 (1.2)	6 (1.6)	57 (3.7)
Prior pregnancy	145 (10.9)	1,186 (9.5)	113 (29.1)	544 (35.7)
Uterine fibroids	8 (0.6)	34 (0.3)	7 (1.8)	41 (2.7)
Uterine polyp	0 (0.0)	11 (0.1)	0 (0.0)	NR
Endometriosis	11 (0.8)	63 (0.5)	9 (2.3)	47 (3.1)
Pelvic infections	12 (0.9)	65 (0.5)	13 (3.4)	54 (3.5)
Uterine prolapse	NR	12 (0.1)	2 (0.5)	5 (0.3)
Uterine or cervical cancer	0 (0.0)	NR	0 (0.0)	NR

CPRD, Clinical Practice Research Datalink; HES, Hospital Episode Statistics; HMB, heavy menstrual bleeding; NA, not applicable; NR, not reportable (counts <5 patients); SD, standard deviation; VWD, von Willebrand disease.

The reasons for censoring comprised end of record (VWD: 65.5%; non-VWD matches: 83.6%), having an HMB code (VWD: 27.1%; non-VWD matches: 11.3%), having a hysterectomy code (VWD: 3.0%; non-VWD matches: 1.2%), reaching the age of 65 years (VWD: 3.8%; non-VWD matches: 3.1%), and death (VWD: 0.6%; non-VWD matches: 0.7%). The mean ± standard deviation (SD) duration of follow-up for HMB was 7.2 ± 6.3 years for women with VWD and 8.6 ± 6.9 years for women without VWD.

HMB was recorded after cohort entry in 388 of 1,335 (29.1%) women with VWD and 1,524 of 12,463 (12.2%) women without VWD (Table 1). The median [range] age at first HMB after cohort entry among women with VWD was lower (31.0 [10–55] years) than among women without VWD (36.5 [10–59] years). Pregnancy was recorded before the HMB index date in 29.1% of women with VWD, compared with 35.7% of women without VWD. The number of individuals with other covariates was low and was balanced between women with and without VWD.

**FIG. 1.**
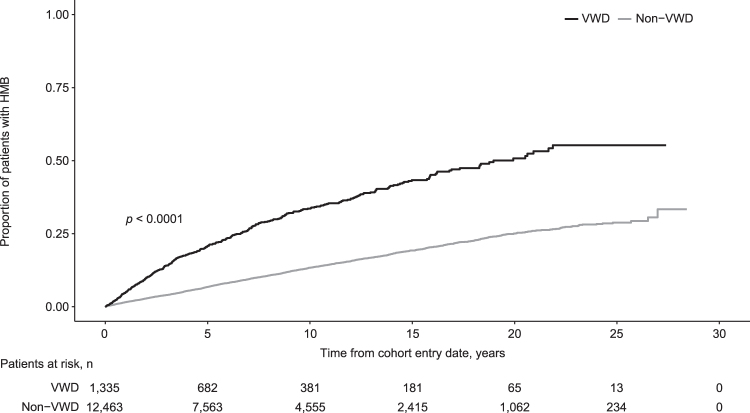
Cumulative incidence of HMB after cohort entry date in women with or without VWD. HMB, heavy menstrual bleeding; VWD, von Willebrand disease.

The cumulative incidence of HMB after cohort entry was elevated in women with VWD compared with women without VWD (Fig. 1; *p* < 0.0001). This trend was observed across all age groups (at cohort entry date; *p* < 0.0001; Supplementary Fig. S1) and regardless of prior HMB status (*p* < 0.001; Supplementary Fig. S2).

The IR for HMB was 40.2 (95% CI: 36.3 − 44.4) per 1,000 patient-years for women with VWD and 14.2 (95% CI: 13.5 − 14.9) per 1,000 patient-years for women without VWD (Table 2). The IRR for HMB was nearly three times higher for women with versus without VWD (Table 2). For women either with and without VWD, IRs for HMB varied by age: women with VWD had higher IRs than women without VWD across all age groups, and the biggest differences in rates were observed in the younger age groups (Table 2).

**Table 2.** Incidence Rates and Incidence Rate Ratios of Heavy Menstrual Bleeding in Women With or Without von Willebrand Disease

	Women with VWD			Women without VWD			IRR^a^	
	HMB cases	PY	IR/1,000 PY (95% CI)	HMB cases	PY	IR/1,000 PY (95% CI)	Crude IRR^a^ (95% CI)	Adj. IRR^a^ (95% CI)
Overall	388	9,644	40.2 (36.3 − 44.4)	1,524	107,567	14.2 (13.5 − 14.9)	2.84 (2.54 − 3.17)	2.74 (2.44 − 3.07)^b^
By age group, years								
10 − 19	93	2,136	43.5 (35.1 − 53.4)	209	23,076	9.1 (7.9 − 10.4)	4.81 (3.77 − 6.14)	4.64 (3.63 − 5.94)^c^
20 − 29	83	2,327	35.7 (28.4 − 44.2)	262	26,041	10.1 (8.9 − 11.4)	3.55 (2.77 − 4.54)	3.20 (2.48 − 4.13)^c^
30 − 39	105	2,284	46.0 (37.6 − 55.7)	439	26,270	16.7 (15.2 − 18.3)	2.75 (2.22 − 3.40)	2.53 (2.03 − 3.14)^c^
40 − 49	91	1,645	55.3 (44.5 − 67.9)	525	19,699	26.7 (24.4 − 29.0)	2.08 (1.66 − 2.59)	1.96 (1.57 − 2.46)^c^
50 − 64	16	1,253	12.8 (7.3 − 20.7)	89	12,481	7.1 (5.7 − 8.8)	1.79 (1.05 − 3.05)	1.65 (0.96 − 2.84)^c^
By prior HMB								
No prior HMB	341	9,082	37.5 (33.7 – 41.7)	1,468	106,195	13.8 (13.1 – 14.5)	2.72 (2.41 – 3.06)	2.78 (2.47 – 3.13)^d^
Prior HMB	47	562	83.7 (61.5 – 111.3)	56	1,372	40.8 (30.8 – 53.0)	2.05 (1.39 – 3.02)	2.00 (2.35 – 2.96)^d^

^a^ IRR of HMB for VWD compared with non-VWD (reference).

^b^ IRR adjusted for age and prior HMB.

^c^ IRR adjusted for prior HMB.

^d^ IRR adjusted for age.

Adj, adjusted; CI, confidence intervals; IR, incidence rate; IRR, incidence rate ratio; PY, patient-years.

### Hysterectomy

In total, 1,374 women with VWD and 12,791 matched women without VWD were included in the hysterectomy analyses (Table 3; Supplementary Table S2). Most women with VWD (59.8%) or without VWD (59.4%) had both CPRD GOLD data and eligibility for HES linkage. Patient characteristics in both cohorts were similar with regard to age at cohort entry (median [range]: 26.0 [10–89] and 26.0 [10–91] years, respectively) and length of follow-up. A history of HMB before cohort entry was recorded in 9.0% of women with VWD compared with 1.9% of women without VWD. The reasons for censoring comprised end of record (VWD: 91.3%; non-VWD matches: 95.3%), having a hysterectomy code (VWD: 6.4%; non-VWD matches: 2.5%), and death (VWD: 2.3%; non-VWD matches: 2.2%). The mean ± SD duration of follow-up for hysterectomy was 9.3 ± 7.2 years for women with VWD and 9.6 ± 7.4 years for women without VWD.

**Table 3.** Characteristics of the Study Population Used for the Hysterectomy Analysis: Women With or Without von Willebrand Disease Overall and Those with Hysterectomy Recorded After Cohort Entry Date

	All patients in hysterectomy analysis		Women undergoing hysterectomy after cohort entry	
	Women with VWD (*n* = 1,374)	Women without VWD (*n* = 12,791)	Women with VWD (*n* = 88)	Women without VWD (*n* = 320)
Data source, *n* (%)	Cohort entry		Hysterectomy	
CPRD data plus eligible for HES linkage	821 (59.8)	7,599 (59.4)	—	—
CPRD data only	553 (40.3)	5,192 (40.6)	66 (75.0)	233 (72.8)
HES data only	—	—	NR	NR
CPRD plus HES data	—	—	21 (23.9)	83 (25.9)
Year, *n* (%)	Cohort entry		Hysterectomy	
1988 − 1999	559 (40.7)	5,202 (40.7)	37 (42.0)	108 (33.8)
2000 − 2009	622 (45.3)	5,831 (45.6)	31 (35.2)	136 (42.5)
2010 − 2016	193 (14.1)	1,758 (13.7)	20 (22.7)	76 (23.8)
Age, years, *n* (%)	Cohort entry		Hysterectomy	
10 − 29	820 (59.7)	7,784 (60.9)	5 (5.7)	NR
30 − 39	299 (21.8)	2,862 (22.4)	32 (35.4)	75 (23.4)
40 − 49	147 (10.7)	1,295 (10.1)	30 (34.1)	159 (49.7)
≥50	108 (7.9)	850 (6.6)	21 (23.9)	83 (25.9)
Mean (SD) age, years	27.6 (15.0)	27.1 (14.3)	43.2 (10.9)	46.0 (9.6)
Median (range) age, years	26.0 (10 – 89)	26.0 (10 – 91)	41.0 (27 – 81)	44.5 (28 – 80)
Covariates, *n* (%)	Before cohort entry		Before hysterectomy date	
Hypertension	40 (2.9)	238 (1.9)	12 (13.6)	39 (12.2)
Diabetes	10 (0.7)	95 (0.7)	NR	14 (4.4)
Hypercholesterolemia	NR	51 (0.4)	NR	11 (3.4)
Chronic kidney disease	NR	17 (0.1)	NR	8 (2.5)
Liver disease	NR	9 (0.1)	0 (0.0)	0 (0.0)
Alcohol abuse	23 (1.7)	141 (1.1)	NR	9 (2.8)
Drug abuse	9 (0.7)	69 (0.5)	0 (0.0)	NR
Thyroid disorder	12 (0.9)	166 (1.3)	NR	24 (7.5)
Prior pregnancy	145 (10.6)	1,187 (9.3)	28 (31.8)	73 (22.8)
HMB	124 (9.0)	240 (1.9)	46 (52.3)	157 (49.1)
Uterine fibroids	9 (0.7)	36 (0.3)	17 (19.3)	45 (14.1)
Uterine polyp	0 (0.0)	11 (0.1)	NR	NR
Endometriosis	11 (0.8)	62 (0.5)	12 (13.6)	35 (10.9)
Pelvic infections	12 (0.9)	65 (0.5)	NR	27 (8.4)
Uterine prolapse	NR	15 (0.1)	NR	37 (11.6)
Uterine or cervical cancer	0 (0.0)	NR	NR	10 (3.1)

Hysterectomy was recorded in 88 women with VWD (6.4%) and 320 women without VWD (2.5%) after cohort entry (Table 3). Most women were aged 30–59 years at the hysterectomy date; however, the median [range] age at hysterectomy was lower for women with VWD (41.0 [27–81] years) than for women without VWD (44.5 [28–80] years). A higher proportion of women with VWD (31.8%) who had a hysterectomy record also had a prior pregnancy record than did women without VWD (22.8%).

**FIG. 2.**
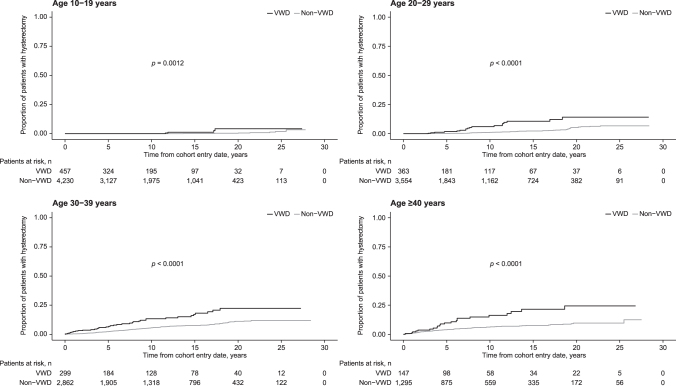
Cumulative incidence of hysterectomy in women with or without VWD, stratified by age at cohort entry date.

The cumulative incidence of hysterectomy after the cohort entry date was significantly higher in women with VWD than in women without VWD (*p* < 0.0001). When stratified by age, the cumulative incidence was higher for women aged ≥30 years at cohort entry than for those aged 10–29 years at cohort entry (Fig. 2).

The IR for hysterectomy was 6.9 (95% CI: 5.5–8.5) per 1,000 patient-years for women with VWD and 2.6 (95% CI: 2.3–2.9) per 1,000 patient-years for women without VWD (Table 4). The IRR for hysterectomy was more than two times higher for women with versus without VWD (Table 4). Although IRs for hysterectomy varied by age, women with VWD had higher IRs than women without VWD in all age groups (Table 4). IRRs were higher in women with VWD than in women without VWD, regardless of prior history of HMB (Table 4).

**Table 4.** Incidence Rates and Incidence Rate Ratios of Hysterectomy in Women With or Without von Willebrand Disease

	Women with VWD			Women without VWD			IRR^a^	
	Hysterectomy cases	PY	IR/1,000 PY (95% CI)	Hysterectomy cases	PY	IR/1,000 PY (95% CI)	Crude IRR^a^ (95% CI)	Adj. IRR^a^ (95% CI)
Overall	88	12,751	6.9 (5.5 − 8.5)	320	123,419	2.6 (2.3 − 2.9)	2.66 (2.12 − 3.65)	2.14 (1.69 − 2.71)^b^
By age group, years								
10 − 29	5	5,295	0.9 (0.3 − 2.2)	NR	51,210	0.1 (0.0 − 0.2)	Not calculated^c^	Not calculated^c^
30 − 39	32	2,819	11.3 (7.8 − 16.0)	75	28,496	2.6 (2.1 − 3.3)	4.31 (2.85 − 6.52)	3.58 (2.36 − 5.44)^d^
40 − 49	30	2,259	13.3 (9.0 − 19.0)	159	23,030	6.9 (5.9 − 8.1)	1.92 (1.30 − 2.84)	1.57 (1.06 − 2.33)^d^
≥50	21	2,378	8.8 (5.5 − 13.5)	83	20,683	4.0 (3.2 − 5.0)	2.20 (1.36 − 3.55)	2.21 (1.37 − 3.57)^d^
By prior HMB								
No prior HMB	42	1,959	21.4 (15.5 − 29.0)	163	10,432	15.6 (13.3 − 18.2)	1.37 (0.98 − 1.93)	1.78 (1.27 − 2.51)^e^
Prior HMB	46	10,782	4.3 (3.1 − 5.7)	157	112,987	1.4 (1.2 − 1.6)	3.07 (2.21 − 4.26)	3.08 (2.22 − 4.28)^e^

^a^ IRR of hysterectomy for VWD compared with non-VWD (reference).

^b^ IRR adjusted for age and prior HMB.

^c^ Too few cases to calculate a stable IRR.

^d^ IRR adjusted for prior HMB.

^e^ IRR adjusted for age.

The HR and cause-specific HR were estimated for hysterectomy, adjusting for competing risk of death. There was no difference between the HR (2.67; 95% CI: 2.11 − 3.39; Schoenfeld residuals test [proportionality assumption]: *p* = 0.096) and cause-specific HR estimate (2.66; 95% CI: 2.10 − 3.38), indicating that there was no difference in the risk of death between women with or without VWD.

## Discussion

In this retrospective analysis of the UK CPRD and HES databases, women with VWD in the general practice setting were more than twice as likely to have HMB or to undergo hysterectomy than women without VWD. HMB was more common in women with versus without VWD, irrespective of age or prior HMB status, although the most substantial difference in rates between the VWD and non-VWD cohorts was observed in younger age groups (10–29 years). The most substantial difference in hysterectomy rates was observed in the 30–39 years age group, with women with VWD in this age group being four times more likely to undergo hysterectomy than those without VWD. Overall, women with VWD tended to have hysterectomy at an earlier age compared with women without VWD.

These findings are in line with those reported in the literature: HMB is reported in 50%–93% of women with a diagnosis of VWD,^7,13,14^ while 5%–24% of girls and women presenting with HMB are diagnosed with VWD.^15–17^ This may, however, be an underestimate of the prevalence of VWD in women with HMB, given the low screening rates reported in the literature, including in women undergoing hysterectomy for HMB.^18,19^ The higher rate of hysterectomy in women with versus without VWD, reported in this analysis as well as other studies,^7,10^ is a concern, especially because women with VWD appear to be at risk of hysterectomy earlier than those without VWD and during key years for fertility and conception (30–39 years).

In addition, the present analysis highlights important unmet needs for women with VWD in the general practice setting. Although the analyzed population managed in a GP setting would be expected to have milder disease than that observed from a specialist setting, these findings are in line with previous studies that reported high incidences of HMB and hysterectomy among women visiting hemophilia treatment centers.^7,9,10^ For example, a cross-sectional study was conducted in the Netherlands in women with moderate and severe VWD recruited at hemophilia treatment centers.^7^ HMB was reported in 81% of women and 20% underwent a hysterectomy mainly because of HMB. In addition, >50% of women with VWD in this study experienced bleeding complications following childbirth or pregnancy loss.^7^ HMB was also reported in 78% of women in a U.S. surveillance study of patients seeking care at hemophilia treatment centers, and 11% of women in this study had undergone hysterectomy to control HMB.^9^

The international guidelines for VWD management recommend the use of either hormonal therapy or tranexamic acid in women with VWD and HMB, depending on the woman's reproductive needs.^5^ Long-term prophylaxis with VWF replacement therapy has been conditionally recommended for patients with VWD who have a history of severe and frequent bleeds. Long-term VWF prophylaxis has been shown to prevent recurrent bleeding episodes^5,20^ and thus could potentially reduce severe/frequent HMB and, consequently, the need for hysterectomy in women with VWD, especially those of reproductive age. However, questions have been raised regarding the applicability of VWF prophylaxis for HMB because of the limited number of women in the prophylaxis studies evaluated by the guideline panel, although no differences were seen between men and women for other bleed types. The guideline panel has, therefore, recommended additional trials to evaluate the use of VWF prophylaxis for HMB.^5^

The analysis reported herein used a large database from the UK, where there is universal free health care for UK legal residents and consequently few barriers to care.^11^ Therefore, the study is likely to have captured all the women with VWD covered in the CPRD GOLD database who had a VWD diagnosis at any time. The UK CPRD database has several positive features, including well-documented validity of recorded information,^21–23^ long average follow-up time, and a relatively stable population. As the data were recorded by GPs, this research provides a description of the experience, diagnoses, and care of people with a broad range of VWD severities. By contrast, much of the published literature on VWD to date describes patients who attend hematology clinics or receive other specialty care and who therefore may have more severe VWD.

It should also be noted that in this analysis women with VWD were matched with women who did not have VWD, based on age, calendar year, and the general practice they attended. This was done to control for differences in the distribution of key patient characteristics, including age, HES linkage eligibility, geography, socioeconomic factors, medical care, and surveillance bias (*e.g.*, variable data recording practices and HES linkage eligibility). Combined data from CPRD (primary care data from the UK) and HES (hospital data from England) were used to capture as many outcomes as possible; in particular, CPRD GOLD provided long-term data for most patients, while the availability of HES linkage (inpatient, outpatient, and emergency care) supplemented details about hospitalizations for 59% of women with VWD and non-VWD matches.

One of the main limitations of this study is the possible underestimation of HMB: it is not possible to evaluate repeated episodes of HMB owing to variability in the recording of bleeding episodes in the CPRD. Most women had only one code for HMB, which may have been because GPs recorded this diagnosis one time only, not at each occurrence. The increased cumulative incidences of HMB observed in this analysis may, at least in part, be the result of increased surveillance of patients with VWD compared with matched patients without VWD. However, this is unlikely to affect the results for hysterectomy, as this is a major surgical event that is likely to be captured by GPs. Moreover, HES data were used to improve the capture of HMB and hysterectomies for CPRD GOLD patients in England where linkage was available (∼60% of study patients). Finally, it should be noted that CPRD does not capture VWD care conducted in a secondary/specialist care setting unless the GP records the encounter in the patient's electronic medical record. The results of this study should, therefore, be interpreted with this caveat in mind; one should not assume that patients are not receiving proper care based solely on diagnoses recorded by the GP.

In conclusion, this retrospective UK general practice population-based matched cohort analysis describes real-world health outcomes for a broad population of women with VWD and was not limited to women seen in specialist centers, who are more likely to have moderate or severe VWD. HMB and hysterectomy were more common in women with VWD than in women without VWD, and tended to be recorded at an earlier age in women with versus without VWD. These findings highlight the substantial impact of VWD on women's health, especially during the key years for fertility and conception.

## Supplementary Material

Supplemental data

Supplemental data

Supplemental data

Supplemental data

Supplemental data
