# Supplementary material for: Impact of von Willebrand Disease on Women's Health Outcomes: A Matched Cohort Database Study
Source: J Womens Health (Larchmt). 2022 Sep 15;31(9):1262–70. doi: 10.1089/jwh.2022.0082 (PMC9527044; doi:10.1089/jwh.2022.0082)
Supplement: Supplemental data [file Suppl_AppendixS1.docx]

**SUPPLEMENTAL APPENDIX S1. READ CODES FOR EXCLUDED CONDITIONS.**

| *Read Code* | *Description* |
| --- | --- |
| C154100  C154011  C154600  F395000  M210.11  2377.00  N100.00  N100.11  N045000  G755.00  G755000  G755100  G755200  G755z00  N200.00  G757.00  G757.11  G757.12  AD61.00  K425200  N012000  N012011  J690.00  J690.11  J690.13  J690.14  J690.15  J690.16  J690.17  J690.13  J690z00  J690000  J690100  13B2.00  8B55.00  ZC2C200  F370000  D310.00  D310000  D310011  D310012  D310z00  K032600  K032y13  K0A2300  K0A3300  Kyu0900  J4...12  J40..00  J40..11  J40..12  J400.00  J400000  J400100  J400200  J400300  J400400  J400z00  J401.00  J401000  J401100  J401z00  J401z11  J402.00  J40z.00  J41..00  J41..11  J41..12  J410.00  J410000  J410100  J410200  J410300  J410z00  J411.00  J412.00  J41y.00  J41y000  J41y100  J41yz00  J41z.00  Jyu4000  Jyu4100  N031000  N031100  N045400  J08z900  14C4.11  M210200  F371000  F396100  H57y400  K01x400  K01x411  M154.00  M154000  M154100  M154200  M154300  M154400  M154500  M154600  M154z00  N000.00  N000000  N000300  N000400  N000z00  Nyu4300  N000100  M154700  F371000  H57y400  K01x400  K01x411  F20..00  F20..11  F200.00  F201.00  F202.00  F203.00  F204.00  F206.00  F207.00  F208.00  F20z.00  F21..00  666A.00  666B.00  8CS1.00  8Cc0.00  8Cc1.00  8Cc2.00  8Cc3.00  8Cc4.00  9kG..00  F21X.00  F21y.00  F21yz00  F21z.00  Fyu4.00  Fyu4000  Fyu4100  Fyu4200  F380.00  F380100  F380z00  M145.00  M145000  M145z00  F4Cy100  M146.00  M146000  M146011  M146100  M146z00  Myu1200  M142.11  M144.00  M144000  M144200  M144300  M144500  M144600  M144700  M144z00  M145100  G75..00  G750.00  G759.00  F371100  F396300  G75z.00  K01x300  G758.00  G751000  G751z00  N20..00  N20..11  N200.00  A993.00  A993.11  N011.12  N011.00  N011x00  N011z00  N013.00  N013700  N01w.00  N01w000  N01w500  N01w600  N01w900  N01wB00  N01wD00  N01wK00  N038.00  AD5..00  AD50.00  AD51.00  AD52.00  AD53.00  AD54.00  AD55.00  G558300  F013.00  F326300  F374900  F396500  H57y200  J63A.00  N233200  M210.00  M210.11  M210300  N001.11  N001.12  N001000  M210400  M210500  M210z00  N001.00  N001100  F396600  N002.00  N002.11  F396700  H57y300  N04y200  N005.00  N043.00  D313.00  D313.11  42P2.11  D313.12  D313.14  D313.15  D313000  D313011  D313012  D313200  F4E5311  M295100  M295.00  M295000  M295z00  G754.00  N006.00  J614111  C020.11  C020.12  F4G2000  F4G2100  F4G2z00  C052.00  C052.11  C052.12  C046.00  C394.00  B6...00  B6...11  B600.00  B600100  B600300  B601.00  B601z00  B602.00  B620.00  B620000  B620100  B620800  B620z00  B624.00  B624.11  B624000  B624300  B627.00  B627000  B627200  B627300  B627500  B627600  B627700  B627800  B627B00  B627C00  B627C11  B627D00  B627W00  B627X00  B62x.00  A789700  AyuC600  B62x400  B62y.00  B62y000  B62y100  B62y500  B62y600  B62y700  B62y800  B62yz00  B62z.00  B62zz11  ByuDC00  ByuDE00  ByuDF00  ByuDF11  B61..00  B612.00  B613.00  B613z00  B614.00  B614000  B614100  B614200  B614400  B614z00  B615.00  B615200  B616.00  B616400  B61z.00  B61z000  B61z100  B61z200  B61zz00  B62..00  ByuD000  B621.00  B621z00  B622.00  B62x000  B62x100  B62x200  B62xX00  B623.00  B623z00  B625.00  B625.11  B625800  B626.00  B626800  B63..00  B630.00  B630.11  B630.12  B630000  B630100  B630200  B630300  B631.00  B63y.00  B63z.00  N330900  B64..00  B64..11  B640.00  B641.00  B641.11  B64y.00  B64y100  B64y200  B64yz00  B64z.00  B65..00  B650.00  B651.00  B651.11  B651z00  B652.00  B653.00  B653000  B65y100  B65z.00  B66..00  B66..12  B660.00  B661.00  B67..00  B670.00  B671.00  B671.11  B672.00  B673.00  B674.00  B675.00  B67y000  B69..00  B690.00  B691.00  B67z.00  B68..00  B680.00  B681.00  B68y.00  B68z.00  B6y..00  B6y0.00  B6y0.11  B6y1.00  B6z..00  B6z0.00  C330000  C333000  ByuD.00  ByuD500  4C53.00  4C54.00  BBTA.11  BBg..00  BBg0.00  BBg1.00  BBg1.11  BBg1000  BBg2.00  BBg2.11  BBg3.00  BBg4.00  BBg5.00  BBg6.00  BBg7.00  BBg8.00  BBg9.00  BBg9.11  BBg9.12  BBgA.00  BBgA.11  BBgB.00  BBgC.00  BBgC.11  BBgC.12  BBgD.00  BBgE.00  BBgF.00  BBgG.00  BBgG.11  BBgG.12  BBgG.13  BBgH.00  BBgJ.00  BBgJ.11  BBgK.00  BBgL.00  BBgM.00  BBgN.00  BBgP.00  BBgQ.00  BBgR.00  BBgS.00  BBgT.00  BBgV.00  BBgz.00  BBh..00  BBh0.00  BBh0.11  BBh1.00  BBh2.00  BBhz.00  BBj..00  BBj0.00  BBj0.11  BBj1.00  BBj1000  BBj1100  BBj2.00  BBj3.00  BBj4.00  BBj5.00  BBj6.00  BBj6000  BBj6100  BBj6200  BBj7.00  BBj8.00  BBj9.00  BBjA.00  BBjz.00  BBk..00  BBk0.00  BBk0.11  BBk0.12  BBk0.13  BBk0.14  BBk1.00  BBk1.11  BBk1.12  BBk2.00  BBk2.11  BBk3.00  BBk4.00  BBk5.00  BBk6.00  BBk7.00  BBk7.11  BBk8.00  BBkz.00  BBm4.00  BBm5.00  BBr..00  BBr0.00  BBr0000  BBr0100  BBr0111  BBr0112  BBr0113  BBr0200  BBr0300  BBr0400  BBr0z00  BBr1.00  BBr1000  BBr1011  BBr1z00  BBr2.00  BBr2000  BBr2011  BBr2100  BBr2200  BBr2300  BBr2400  BBr2500  BBr2600  BBr2700  BBr2z00  BBr3.00  BBr3000  BBr3z00  BBr4.00  BBr4000  BBr4011  BBr4100  BBr4111  BBr4200  BBr4z00  BBr5.00  BBr5000  BBr5z00  BBr6.00  BBr6000  BBr6011  BBr6012  BBr6100  BBr6200  BBr6300  BBr6311  BBr6400  BBr6500  BBr6600  BBr6700  BBr6800  BBr6900  BBr6z00  BBr7.00  BBr7000  BBr7z00  BBr8.00  BBr8000  BBr8z00  BBr9.00  BBr9000  BBr9011  BBr9012  BBr9100  BBr9200  BBr9300  BBr9400  BBr9z00  BBrA.00  BBrA000  BBrA100  BBrA111  BBrA200  BBrA300  BBrA400  BBrA411  BBrA500  BBrA600  BBrA700  BBrA800  BBrAz00  BBrz.00  BBs..00  BBs2.00  BBs3.00  BBs3.11  BBs4.00  BBs5.00  BBsz.00  BBv..00  BBv0.00  BBv1.00  BBv2.00  BBn..00  BBn0.00  BBn0.11  BBn0.12  BBn0.13  BBn0.14  BBn1.00  BBn1.11  BBn2.00  BBn2.11  BBn2.12  BBn3.00  BBnz.00  BBp..00  BBp0.00  BBp1.00  BBp2.00  BBpz.00  BBq..00  BBq0.00  BBqz.00  BBmH.00  B934.00  B934.11  B934.12  BBs0.00  BBs0.11  D410.00  D410000  D410011  D410100  D410200  D410300  D410400  D410z00  D416.00  Q454.00  Q454000  Q454100  Q454z00  42P2.00  42P2.11  42P3.00  B937.12  B937400  B937411  B937500  BBs4.00  D312.00  D312z00  D313.00  D313.11  D313.12  D313.13  D313.14  D313.15  D313000  D313011  D313012  D313100  D313111  D313200  D313211  D313300  D313y00  D313z00  D313z11  D314.00  D314000  D314011  D314100  D314200  D314300  D314y00  D314z00  D314z11  D315.00  D3y0.00  D3y1.00  D3y1.11  G756100  B675.00  B677.00  D41y100  143..11  1431.00  1431.11  1432.00  1433.00  C0...00  C02..00  C02..11  C02..12  C02zz00  C03..00  C04..00  C04..12  C04..13  C040.00  C040.11  C041.00  C041000  C041z00  C042.00  C043.00  C043000  C043100  C043200  C043z00  C044.00  C045.00  C046.00  C047.00  C04y.00  C04z.00  C04z.11  C04z.12  C04z.13 | Addison's disease  Addisonian crisis  Addisonian crisis  Myopathy due to Addison's disease  Addison's keloid  On examination – ankylosing spondylitis chest deformity  Ankylosing spondylitis  Marie-Strumpell spondylitis  Juvenile ankylosing spondylitis  Giant cell arteritis  Cranial arteritis  Temporal arteritis  Horton's disease  Giant cell arteritis NOS  Giant cell arteritis with polymyalgia  Takayasu's disease  Aortic arch arteritis  Pulseless disease  Behcet's syndrome  Ulceration of vulva in Behcet's disease  Arthropathy in Behcet's syndrome of unspecified site  Behcet's syndrome arthropathy  Coeliac disease  Coeliac rickets  Gluten enteropathy  Sprue – nontropical  Steatorrhea – idiopathic  Villous atrophy  Partial villous atrophy  Gluten enteropathy  Coeliac disease NOS  Congenital coeliac disease  Acquired coeliac disease  Gluten free diet  Gluten-free diet  Dietary advice for coeliac disease  Guillain-Barre syndrome  Allergic purpura  Henoch-Schonlein purpura  Anaphylactoid purpura  Acute vascular purpura  Allergic purpura NOS  Berger's IgA or IgG nephropathy  Mesangioproliferative glomerulonephritis NEC  Recurrent and persistent haemuria with diffuse mesangial proliferative glomerulnephritis  Chronic nephritic syndrome, diffuse mesangial proliferative glomerulonephritis  Unspecified nephritic syndrome, diffuse mesangial proliferative glomerulonephritis  Inflammatory bowel disease  Regional enteritis – Crohn's disease  Crohn's disease  Granulomatous enteritis  Regional enteritis of the small bowel  Regional enteritis of the duodenum  Regional enteritis of the jejunum  Crohn's disease of the terminal ileum  Crohn's disease of the ileum unspecified  Crohn's disease of the ileum NOS  Crohn's disease of the small bowel NOS  Regional enteritis of the large bowel  Regional enteritis of the colon  regional enteritis of the rectum  Crohn's disease of the large bowel NOS  Crohn's colitis  Regional ileocolitis  Regional enteritis NOS  Idiopathic proctocolitis  Mucous colitis and/or proctitis  Ulcerative colitis and/or proctitis  Ulcerative proctocolitis  Ulcerative ileocolitis  Ulcerative colitis  Ulcerative rectosigmoiditis  Ulcerative proctitis  Ulcerative proctocolitis NOS  Ulcerative (chronic) enterocolitis  Ulcerative (chronic) ileocolitis  Other idiopathic proctocolitis  Pseudopolyposis of colon  Toxic megacolon  Other idiopathic proctocolitis NOS  Idiopathic proctocolitis NOS  Other Crohn's disease  Other ulcerative colitis  Arthropathy in ulcerative colitis  Arthropathy in Crohn's disease  Juvenile arthritis in ulcerative colitis  Orofacial Crohn's disease  History of ulcerative colitis  Lichen sclerosus et atrophicus  Polyneuropathy in disseminated lupus erythematosus  Myopathy due to disseminated lupus erythematosus  Lung disease with systemic lupus erythematosus  Nephrotic syndrome in systemic lupus erythematosus  Lupus nephritis  Lupus erythematosus  Lupus erythematosus chronicus  Discoid lupus erythematosus  Lupus erythematosus migrans  Lupus erythematosus nodularis  Lupus erythematosus profundus  Lupus erythematosus tumidus  Lupus erythematosus unguium mutilans  Lupus erythematosus NOS  Systemic lupus erythematosus  Disseminated lupus erythematosus  Systemic lupus erythematosus with organ or systemic involvement  Systemic lupus erythematosus with pericarditis  Systemic lupus erythematosus nos  Other forms of systemic lupus erythematosus  Libman-Sacks disease  Subacute cutaneous lupus erythematosus  Polyneuropathy in disseminated lupus erythematosus  Lung disease with systemic lupus erythematosus  Nephrotic syndrome in systemic lupus erythematosus  Lupus nephritis  Multiple sclerosis  Disseminated sclerosis  Multiple sclerosis of the brain stem  Multiple sclerosis of the spinal cord  Generalised multiple sclerosis  Exacerbation of multiple sclerosis  Benign multiple sclerosis  Primary progressive multiple sclerosis  Relapsing and remitting multiple sclerosis  Secondary progressive multiple sclerosis  Multiple sclerosis NOS  Other central nervous system demyelinating diseases  Multiple sclerosis review  Multiple sclerosis multidisciplinary review  Multiple sclerosis care plan agreed  Management of multiple sclerosis in onset phase  Management of multiple sclerosis in early disease phase  Management of multiple sclerosis in stable disability phase  Management of MS in progressive disability phase  Management of multiple sclerosis in palliative phase  Specialist service for patient with multiple sclerosis - enhanced service administration  Acute disseminated demyelination; unspecified  Other specified central nervous system demyelinating disease  Other specified central nervous system demyelination NOS  Central nervous system demyelination NOS  Demyelinating diseases of the central nervous system  Other specified acute disseminated demyelination  Other specified demyelinating diseases/the CNS  Acute disseminated demyelination; unspecified  Myasthenia gravis  Juvenile or adult myasthenia gravis  Myasthenia gravis NOS  Pemphigoid  Bullous pemphigoid  Pemphigoid NOS  Ocular pemphigoid  Benign mucous membrane pemphigoid  Benign mucous membrane pemphigoid with no eye involvement  Cicatricial pemphigoid  Ocular pemphigoid  Benign mucous membrane pemphigoid NOS  Other pemphigoid  Juvenile pemphigoid  Pemphigus  Benign pemphigus  Erythematous pemphigus  Foliaceous pemphigus  Pemphigus vegetans  Pemphigus vulgaris  Wildfire pemphigus  Pemphigus NOS  Benign pemphigus NOS  Polyarteritis nodosa and allied conditions  Polyarteritis nodosa  Juvenile polyarteritis  Polyneuropathy in polyarteritis nodosa  Myopathy due to polyarteritis nodosa  Polyarteritis nodosa and allied conditions NOS  Nephrotic syndrome in polyarteritis nodosa  Churg-Strauss vasculitis  Kawasaki disease  Acute febrile mucocutaneous lymph node syndrome NOS  Polymyalgia rheumatica  Polymyalgia  Giant cell arteritis with polymyalgia  Reiter's disease / syndrome  Reiter's syndrome  Arthropathy in Reiter's disease  Sexually acquired reactive arthropathy  Sexually acquired reactive arthropathy of multiple sites  Sexually acquired reactive arthropathy NOS  Postdysenteric reactive arthropathy  Postdysenteric reactive arthropathy of the ankle and foot  Reactive arthropathy, unspecified  Reactive arthropathy of shoulder  Reactive arthropathy of wrist  Reactive arthropathy of MCP joint  Reactive arthropathy of hip  Reactive arthropathy of knee  Reactive arthropathy of ankle  Reactive arthropathy of IP joint of toe  Reactive arthropathies  Sarcoidosis  Sarcoidosis of lung  Sarcoidosis of lymph nodes  Sarcoidosis of lung with sarcoidosis of lymph nodes  Sarcoidosis of skin  Sarcoidosis of inferior turbinates  Sarcoid arthropathy  Sarcoid heart disease  Meningitis due to sarcoidosis  Multiple cranial nerve palsies in sarcoidosis  Polyneuropathy in sarcoidosis  Myopathy due to sarcoidosis  Pulmonary sarcoidosis  Hepatic granulomas in sarcoidosis  Myositis in sarcoidosis  Circumscribed scleroderma  Addison's keloid  Morphoea  Acrosclerosis  Systemic sclerosis  Progressive systemic sclerosis  Linear scleroderma  Sclerodactyly (= Acrosclerosis)  Circumscribed scleroderma NOS  Scleroderma  CREST syndrome  Myopathy due to scleroderma  Sicca (Sjogren's) syndrome  Keratoconjunctivitis sicca  Myopathy due to Sjogren's disease  Lung disease with Sjogren's disease  Adult-onset Still's disease  Adult Still's Disease  Juvenile rheumatoid arthritis – Still's disease  Primary thrombocytopenia  Evan's syndrome  Auto-immune thrombocytopenia  Idiopathic thrombocytopenic purpura  Megakaryocytic hypoplasia  Thrombocytopenic purpura  Idiopathic thrombocytopenic purpura  Idiopathic purpura  ITP – Idiopathic thrombocytopenic purpura  Thrombocytopenic purpura with absent radius  Vitiligo of eyelid  Vitiligo  Leucoderma  Leucoderma aestivale  Leucoderma NOS  Wegener's granulomatosis  Antiphospholipid syndrome  Autoimmune chronic active hepatitis  Basedow's disease  Graves' disease  Thyrotoxic exophthalmos  Exophthalmic ophthalmoplegia  Endocrine exophthalmos NOS  Chronic lymphocytic thyroiditis  Autoimmune thyroiditis  Hashimoto's disease  Autoimmune myxoedema  Autoimmune disease NOS  Malignant neoplasm of lymphatic and haemopoietic tissue  Malignant neoplasm of histiocytic tissue  Reticulosarcoma  Reticulosarcoma of lymph nodes of head, face and neck  Reticulosarcoma of intra-abdominal lymphnodes  Lymphosarcoma  Lymphosarcoma NOS  Burkitt's lymphoma  Nodular lymphoma (Brill - Symmers disease)  Nodular lymphoma of unspecified site  Nodular lymphoma of lymph nodes of head, face and neck  Nodular lymphoma of lymph nodes of multiple sites  Nodular lymphoma NOS  Leukaemic reticuloendotheliosis  Leukaemic reticuloendotheliosis  Leukaemic reticuloendotheliosis of unspecified sites  Leukaemic reticuloend of intra-abdominal lymph nodes  Non - Hodgkin's lymphoma  Follicular non-Hodgkin's small cleaved cell lymphoma  Follicular non-Hodgkin's large cell lymphoma  Diffuse non-Hodgkin's small cell (diffuse) lymphoma  Diffuse non-Hodgkin mixed sml & lge cell (diffuse) lymphoma  Diffuse non-Hodgkin's immunoblastic (diffuse) lymphoma  Diffuse non-Hodgkin's lymphoblastic (diffuse) lymphoma  Diffuse non-Hodgkin's lymphoma undifferentiated (diffuse)  Other types of follicular non-Hodgkin's lymphoma  Follicular non-Hodgkin's lymphoma  Follicular lymphoma NOS  Diffuse non-Hodgkin's centroblastic lymphoma  Unspecified B-cell non-Hodgkin's lymphoma  Diffuse non-Hodgkin's lymphoma, unspecified  Malignant lymphoma otherwise specified  HIV dis resulting oth types of non-Hodgkin's  HIV disease resulting in other non-Hodgkin's lymphoma  Malignant reticulosis  Malignant lymphoma NOS  Malignant lymphoma NOS of unspecified site  Malignant lymphoma NOS of lymph nodes of head, face and neck  Malignant lymphoma NOS of lymph node inguinal region and leg  Malignant lymphoma NOS of intrapelvic lymph nodes  Malignant lymphoma NOS of spleen  Malignant lymphoma NOS of lymph nodes of multiple sites  Malignant lymphoma NOS  Malignant neoplasms of lymphoid and histiocytic tissue NOS  Immunoproliferative neoplasm  Diffuse non-Hodgkin's lymphoma, unspecified  Unspecified B-cell non-Hodgkin's lymphoma  Non-Hodgkin's lymphoma, unspecified type  Non-Hodgkin's lymphoma NOS  Hodgkin's disease  Hodgkin's sarcoma  Hodgkin's disease, lymphocytic-histiocytic predominance  Hodgkin's, lymphocytic-histiocytic predominance NOS  Hodgkin's disease, nodular sclerosis  Hodgkin's disease, nodular sclerosis of unspecified site  Hodgkin's nodular sclerosis of head, face and neck  Hodgkin's nodular sclerosis of intrathoracic lymph nodes  Hodgkin's nodular sclerosis of lymph nodes of axilla and arm  Hodgkin's disease, nodular sclerosis NOS  Hodgkin's disease, mixed cellularity  Hodgkin's mixed cellularity of intrathoracic lymph nodes  Hodgkin's disease, lymphocytic depletion  Hodgkin's lymphocytic depletion lymph nodes axilla and arm  Hodgkin's disease NOS  Hodgkin's disease NOS, unspecified site  Hodgkin's disease NOS of lymph nodes of head, face and neck  Hodgkin's disease NOS of intrathoracic lymph nodes  Hodgkin's disease NOS  Other malignant neoplasm of lymphoid and histiocytic tissue  Other Hodgkin's disease  Mycosis fungoides  Mycosis fungoides NOS  Sezary's disease  T-zone lymphoma  Lymphoepithelioid lymphoma  Peripheral T-cell lymphoma  Other and unspecified peripheral & cutaneous Tcell lymphomas  Malignant histiocytosis  Malignant histiocytosis NOS  Letterer-Siwe disease  Histiocytosis X (acute, progressive)  Letterer-Siwe disease of lymph nodes of multiple sites  Malignant mast cell tumours  Mast cell malignancy of lymph nodes of multiple sites  Multiple myeloma and immunoproliferative neoplasms  Multiple myeloma  Kahler's disease  Myelomatosis  Malignant plasma cell neoplasm, extramedullary plasmacytoma  Solitary myeloma  Plasmacytoma NOS  Lambda light chain myeloma  Plasma cell leukaemia  Other immunoproliferative neoplasms  Immunoproliferative neoplasm or myeloma NOS  Osteoporosis in multiple myelomatosis  Lymphoid leukaemia  Lymphatic leukaemia  Acute lymphoid leukaemia  Chronic lymphoid leukaemia  Chronic lymphatic leukaemia  Other lymphoid leukaemia  Prolymphocytic leukaemia  Adult T-cell leukaemia  Other lymphoid leukaemia NOS  Lymphoid leukaemia NOS  Myeloid leukaemia  Acute myeloid leukaemia  Chronic myeloid leukaemia  Chronic granulocytic leukaemia  Chronic myeloid leukaemia NOS  Subacute myeloid leukaemia  Myeloid sarcoma  Chloroma  Acute promyelocytic leukaemia  Myeloid leukaemia NOS  Monocytic leukaemia  Monoblastic leukaemia  Acute monocytic leukaemia  Chronic monocytic leukaemia  Other specified leukaemia  Acute erythraemia and erythroleukaemia  Chronic erythraemia  Heilmeyer - Schoner disease  Megakaryocytic leukaemia  Mast cell leukaemia  Acute panmyelosis  Acute myelofibrosis  Lymphosarcoma cell leukaemia  Myelomonocytic leukaemia  Acute myelomonocytic leukaemia  Chronic myelomonocytic leukaemia  Other specified leukaemia NOS  Leukaemia of unspecified cell type  Acute leukaemia NOS  Chronic leukaemia NOS  Other leukaemia of unspecified cell type  Leukaemia NOS  Malignant neoplasm lymphatic or haematopoietic tissue OS  Myeloproliferative disorder  Myeloproliferative disease  Myelosclerosis with myeloid metaplasia  Malignant neoplasm lymphatic or haematopoietic tissue NOS  Kaposi's sarcoma of lymph nodes  Waldenstrom's hypergammaglobulinaemic purpura  Waldenstrom's macroglobulinaemia  Malignant neoplasms of lymphoid, haematopoietic and related tissue  Other lymphoid leukaemia  Bone marrow: myeloma cells  Bone marrow: tumour cells  Multiple haemorrhagic sarcoma  Lymphomas; NOS or diffuse  Lymphomatous tumour; benign  Malignant lymphoma NOS  Lymphoma NOS  Malignant lymphoma; diffuse NOS  Malignant lymphoma; non Hodgkin's type  Non Hodgkins lymphoma  Malignant lymphoma; undifferentiated cell type NOS  Malignant lymphoma; stem cell type  Malignant lymphoma; convoluted cell type NOS  Lymphosarcoma NOS  Malignant lymphoma; lymphoplasmacytoid type  Malignant lymphoma; immunoblastic type  Malignant lymphoma; mixed lymphocytic-histiocytic NOS  Reticulolymphosarcoma NOS  Reticulolymphosarcoma; diffuse  Malignant lymphoma; centroblastic-centrocytic; diffuse  Germinoblastoma; diffuse  Malignant lymphoma; follicular centre cell NOS  Malignant lymphoma; lymphocytic; well differentiated NOS  Lymphocytic lymphoma NOS  Lymphocytic lymphosarcoma NOS  Malignant lymphoma; lymphocytic; intermediate different NOS  Malignant lymphoma; centrocytic  Malignant lymphoma; follicular centre cell; cleaved NOS  Malignant lymphoma; lymphocytic; poorly different NOS  Lymphoblastic lymphosarcoma NOS  Lymphoblastic lymphoma NOS  Lymphoblastoma NOS  Prolymphocytic lymphosarcoma  Malignant lymphoma; centroblastic type NOS  Germinoblastic sarcoma NOS  Malignant lymphoma; follicular centre cell; non-cleaved NOS  Malignant lymphoma; small lymphocytic NOS  Malignant lymphoma; small cleaved cell; diffuse  Malignant lymphoma; lymphocytic; intermediate differentiated; diffuse  Malignant lymphoma; mixed small and large cell; diffuse  Malignant lymphomatous polyposis  Malignant lymphoma; large cell; diffuse NOS  Malignant lymphoma; large cell; cleaved; diffuse  Malignant lymphoma; large cell; noncleaved; diffuse  Malignant lymphoma; small cell; noncleaved; diffuse  Lymphoma; diffuse or NOS  Reticulosarcomas  Reticulosarcoma NOS  Reticulum cell sarcoma NOS  Reticulosarcoma; pleomorphic cell type  Reticulosarcoma; nodular  Reticulosarcoma NOS  Hodgkin's disease  Hodgkin's disease NOS  Lymphogranuloma; malignant  Hodgkin's disease; lymphocytic predominance  Hodgkin;s disease; lymphocytic predominance; diffuse  Hodgkin;s disease; lymphocytic predominance; nodular  Hodgkin's disease; mixed cellularity  Hodgkin's disease; lymphocytic depletion NOS  Hodgkin's disease; lymphocytic depletion; diffuse fibrosis  Hodgkin's disease; lymphocytic depletion; reticular type  Hodgkin's disease; nodular sclerosis NOS  Hodgkin’s disease; nodular sclerosis; lymphocytic predominant  Hodgkin’s disease; nodular sclerosis; mixed cellularity  Hodgkin’s disease; nodular sclerosis; lymphocytic depleted  Hodgkin's disease; nodular sclerosis; cellular phase  Hodgkin's paragranuloma  Hodgkin's granuloma  Hodgkin's sarcoma  Hodgkin's disease NOS  Lymphomas; nodular or follicular  Malignant lymphoma; nodular NOS  Brill-Symmers' disease  Follicular lymphosarcoma NOS  Giant follicular lymphoma  Nodular lymphosarcoma NOS  Malignant lymphoma; mixed lymphocytic-histiocytic; nodular  Reticulolymphosarcoma; follicular  Reticulolymphosarcoma; nodular  Malignant lymphoma; centroblastic-centrocytic; follicular  Germinoblastoma; follicular  Malignant lymphoma; lymphocytic; well differentiated; nodular  Malignant lymphoma; lymphocytic; intermediate different; nodular  Malignant lymphoma; follicular centre cell; cleaved; follicular  Malignant lymphoma; lymphocytic; poorly differentiated; nodular  Malignant lymphoma; centroblastic type; follicular  Germinoblastic sarcoma; follicular  Malignant lymphoma; follicular centre cell; noncleaved; follicular  Lymphoma; nodular or follicular NOS  True histiocytic lymphoma  Peripheral T-cell lymphoma NOS  Leukaemias  Leukaemias unspecified  Leukaemia NOS  Acute leukaemia NOS  Blast cell leukaemia  Blastic leukaemia  Stem cell leukaemia  Subacute leukaemia NOS  Chronic leukaemia NOS  Aleukaemic leukaemia NOS  Leukaemia unspecified; NOS  Compound leukaemias  Compound leukaemia  Mixed leukaemia  Compound leukaemia NOS  Lymphoid leukaemias  Lymphoid leukaemia NOS  Lymphatic leukaemia  Acute lymphoid leukaemia  Subacute lymphoid leukaemia  Chronic lymphoid leukaemia  Aleukaemic lymphoid leukaemia  Prolymphocytic leukaemia  Burkitt's cell leukaemia  Adult T-cell leukaemia/lymphoma  Other lymphoid leukaemia NOS  Plasma cell leukaemias  Plasma cell leukaemia  Plasma cell leukaemia NOS  Erythroleukaemias  Erythroleukaemia  Erythraemic myelosis  Acute erythraemia  Di Guglielmo's disease  Chronic erythraemia  Erythroleukaemia NOS  Lymphosarcoma cell leukaemias  Lymphosarcoma cell leukaemia  Lymphosarcoma cell leukaemia NOS  Myeloid leukaemias  Myeloid leukaemia NOS  Granulocytic leukaemia NOS  Myelosis NOS  Acute myeloid leukaemia  Subacute myeloid leukaemia  Chronic myeloid leukaemia  Naegeli-type monocytic leukaemia  Aleukaemic myeloid leukaemia  Neutrophilic leukaemia  Acute promyelocytic leukaemia  Acute myelomonocytic leukaemia  Chronic myelomonocytic leukaemia  Juvenile myelomonocytic leukaemia  Other myeloid leukaemia NOS  Basophilic leukaemias  Basophilic leukaemia  Basophilic leukaemia NOS  Eosinophilic leukaemias  Eosinophilic leukaemia  Eosinophilic leukaemia NOS  Monocytic leukaemias  Monocytic leukaemia NOS  Histiocytic leukaemia  Schilling-type monocytic leukaemia  Acute monocytic leukaemia  Subacute monocytic leukaemia  Chronic monocytic leukaemia  Aleukaemic monocytic leukaemia  Other monocytic leukaemia NOS  Miscellaneous leukaemias  Mast cell leukaemia  Megakaryocytic leukaemia  Thrombocytic leukaemia  Megakaryocytic myelosis  Myeloid sarcoma  Hairy cell leukaemia  Leukaemic reticuloendotheliosis  Acute megakaryoblastic leukaemia  Acute panmyelosis  Acute myelofibrosis  Leukaemic reticuloendotheliosis  Miscellaneous leukaemia NOS  Leukaemia NOS  Miscellaneous myeloproliferative and lymphoproliferative disorders  Chronic myeloproliferative disease  Myelosclerosis with myeloid metaplasia  Megakaryocytic myelosclerosis  Idiopathic thrombocythaemia  Chronic lymphoproliferative disease  Misc myeloproliferative or lymphoproliferative dis NOS  Myelodysplastic syndrome  Monocytoid B-cell lymphoma  Angioendotheliomatosis  Angiocentric T-cell lymphoma  Plasma cell tumours  Plasma cell myeloma  Multiple myeloma  Myeloma NOS  Myelomatosis  Plasmacytic myeloma  Plasma cell tumour; benign  Plasmacytoma; benign  Plasmacytoma NOS  Monostotic myeloma  Solitary myeloma  Plasma cell tumour; malignant  Plasma cell tumour NOS  Mast cell tumours  Mastocytoma NOS  Mast cell sarcoma  Malignant mastocytosis  Mast cell tumour NOS  Burkitt's tumours  Burkitt's tumour  Burkitt's tumour NOS  Large cell lymphoma  Polycythaemia vera  Polycythaemia rubra vera  Primary polycythaemia  Polycythaemia vera  Polycythaemia rubra vera  Secondary polycythaemia  Stress polycythaemia  Spurious polycythaemia  High altitude polycythaemia  Polycythaemia due to cyanotic heart disease  Polycythaemia due to cyanotic respiratory disease  Renal polycythaemia  Secondary polycythaemia NOS  Familial polycythaemia  Polycythaemia neonatorum  Polycythaemia due to donor twin transfusion  Polycythaemia due to maternal fetal transfusion  Polycythaemia neonatorum NOS  Thrombocytopenia  Auto-immune thrombocytopenia  Thrombocythaemia  Idiopathic thrombocythaemia  Essential (haemorrhagic) thrombocythaemia  Primary thrombocythaemia  Idiopathic thrombocythaemia  Idiopathic thrombocythaemia  Other nonthrombocytopenic purpura  Nonthrombocytopenic purpura NOS  Primary thrombocytopenia  Evan's syndrome  Idiopathic thrombocytopenic purpura  Idiopathic purpura  Megakaryocytic hypoplasia  Thrombocytopenic purpura  Idiopathic thrombocytopenic purpura  Idiopathic purpura  Idiopathic thrombocytopenic purpura  Congenital thrombocytopenic purpura  Hereditary thrombocytopenia NEC  Thrombocytopenic purpura with absent radius  Thrombocytopenia-absent radius syndrome  Essential thrombocytopenia NOS  Other specified primary thrombocytopenia  Primary thrombocytopenia NOS  Essential thrombocytopenia NOS  Secondary thrombocytopenia  Post-transfusion purpura  Thrombocytopenia due to massive blood transfusion  Thrombocytopenia due to drugs  Thrombocytopenia due to extracorporeal circulation of blood  Heparin-induced thrombocytopenia  Other specified secondary thrombocytopenia  Secondary thrombocytopenia NOS  Dilutional thrombocytopenia  Thrombocytopenia NOS  Essential thrombocytosis  Reactive thrombocytosis  Secondary thrombocytosis  Thrombotic thrombocytopenic purpura  Acute myelofibrosis  Myelodysplastic and myeloproliferative disease  Myelofibrosis  History of thyroid disorder  History of hyperthyroidism  History of thyrotoxicosis  History of hypothyroidism  History of thyroid disorder NOS  Disorders of thyroid gland  Thyrotoxicosis  Hyperthyroidism  Toxic goitre  Thyrotoxicosis NOS  Congenital hypothyroidism  Acquired hypothyroidism  Thyroid deficiency  Hypothyroidism  Postsurgical hypothyroidism  Post ablative hypothyroidism  Other postablative hypothyroidism  Irradiation hypothyroidism  Postablative hypothyroidism NOS  Iodine hypothyroidism  Other iatrogenic hypothyroidism  Hypothyroidism resulting from para-aminosalicylic acid  Hypothyroidism resulting from phenylbutazone  Hypothyroidism resulting from resorcinol  Iatrogenic hypothyroidism NOS  Postinfectious hypothyroidism  Acquired atrophy of thyroid  Autoimmune myxoedema  Subclinical hypothyroidism  Other acquired hypothyroidism  Hypothyroidism NOS  Pretibial myxoedema – hypothyroid  Thyroid insufficiency  Hypothyroid goitre; acquired |

Ig, immunoglobin; NEC, not elsewhere classifiable; NOS, not otherwise specific
