# Supplementary material for: Impact of von Willebrand Disease on Women's Health Outcomes: A Matched Cohort Database Study
Source: J Womens Health (Larchmt). 2022 Sep 15;31(9):1262–70. doi: 10.1089/jwh.2022.0082 (PMC9527044; doi:10.1089/jwh.2022.0082)
Supplement: Supplemental data [file Suppl_TableS1.docx]

**SUPPLEMENTAL TABLE S1. CHARACTERISTICS OF WOMEN WITH OR WITHOUT VWD IN THE HMB ANALYSIS: TIMING OF VWD DIAGNOSIS**

|  | *All patients in  HMB analysis* | |
| --- | --- | --- |
|  | *Women  with VWD* *(*n *= 1,335)* | *Women  without VWD*  *(*n *= 12,463)* |
| Timing of VWD diagnosis / match date, *n* (%) |  |  |
| Recorded in historical record | 639 (47.9) | 6,207 (49.8) |
| Recorded in active record | 696 (52.1) | 6,256 (50.2) |
| Year of first VWD diagnosis / match date, *n* (%) | |  |
| <1988 | 178 (13.3) | 1,607 (12.9) |
| 1988–1999 | 459 (34.4) | 4,244 (34.1) |
| 2000–2009 | 530 (39.7) | 4,922 (40.1) |
| 2010–2016 | 168 (12.6) | 1,620 (13.0) |
| Age at first VWD diagnosis / match date, *n* (%) | |  |
| <10 | 255 (19.1) | 2,244 (18.0) |
| 10–19 | 315 (23.6) | 3,017 (24.2) |
| 20–29 | 332 (24.9) | 3,242 (26.0) |
| 30–39 | 239 (17.9) | 2,226 (17.9) |
| 40–49 | 124 (9.3) | 1,149 (9.2) |
| 50–64 | 70 (5.2) | 585 (4.7) |
| Mean (SD), years | 23.7 (14.6) | 23.7 (14.1) |
| Median (range), years | 22 (0–79) | 22 (0–80) |

HMB, heavy menstrual bleeding; SD, standard deviation; VWD, von Willebrand disease.
