# Supplementary figures and images for: Impact of von Willebrand Disease on Women's Health Outcomes: A Matched Cohort Database Study
Source: J Womens Health (Larchmt). 2022 Sep 15;31(9):1262–70. doi: 10.1089/jwh.2022.0082 (PMC9527044; doi:10.1089/jwh.2022.0082)

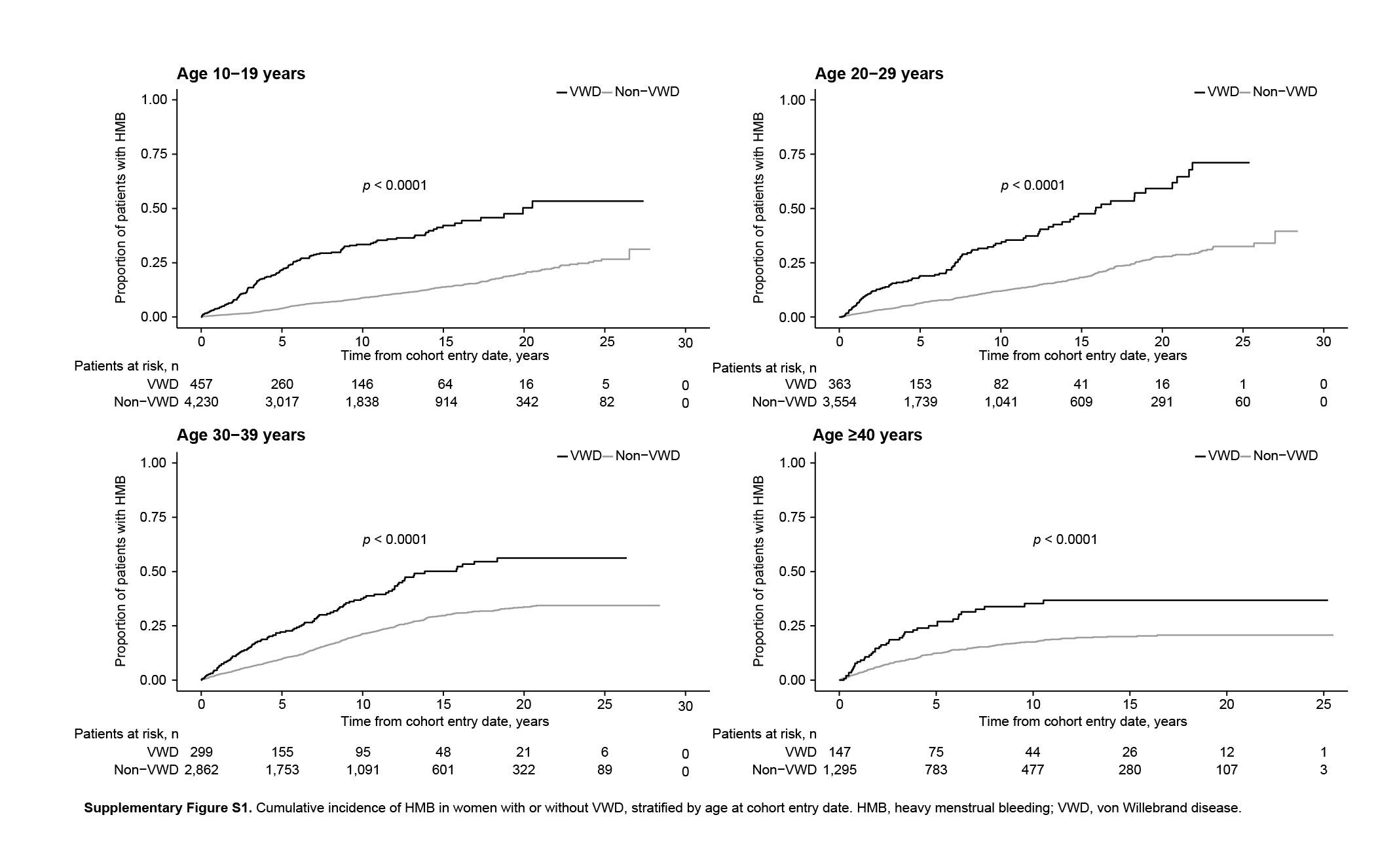

Supplement: Supplemental data [file Suppl_FigS1.jpg]

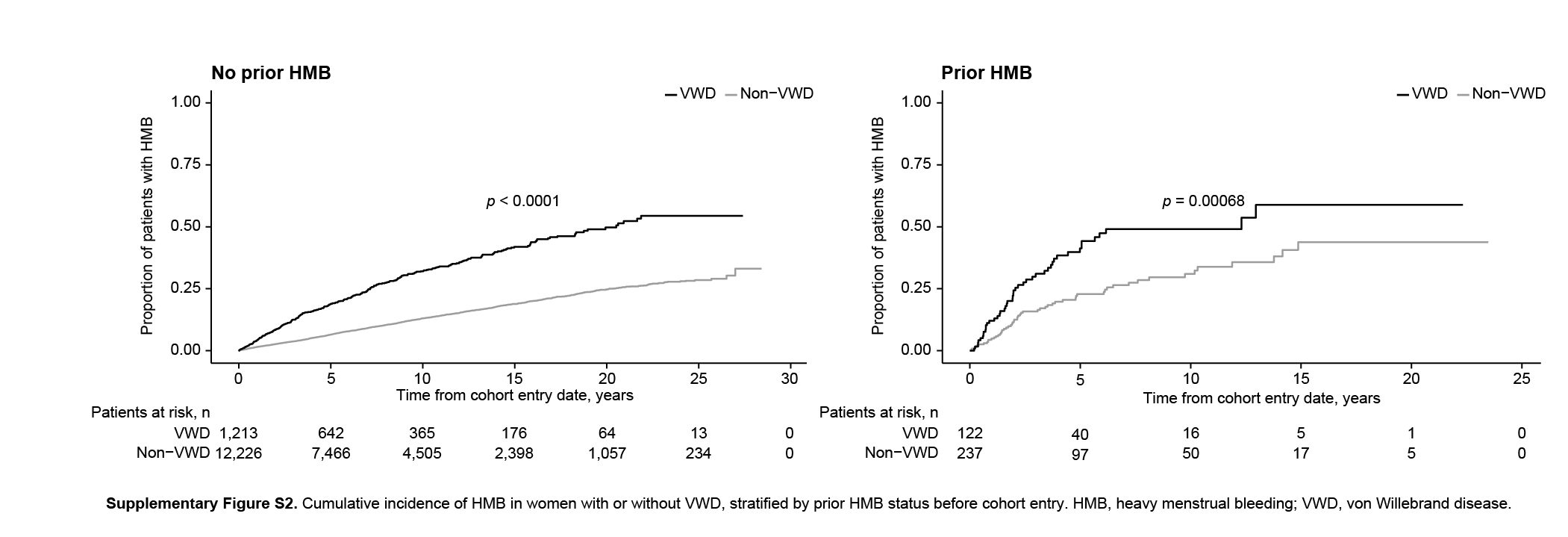

Supplement: Supplemental data [file Suppl_FigS2.jpg]
