# Supplementary material for: Impact of von Willebrand Disease on Women's Health Outcomes: A Matched Cohort Database Study
Source: J Womens Health (Larchmt). 2022 Sep 15;31(9):1262–70. doi: 10.1089/jwh.2022.0082 (PMC9527044; doi:10.1089/jwh.2022.0082)
Supplement: Supplemental data [file Suppl_TableS2.docx]

**SUPPLEMENTAL TABLE S2. CHARACTERISTICS OF WOMEN WITH OR WITHOUT VWD IN THE HYSTERECTOMY ANALYSIS: TIMING OF VWD DIAGNOSIS**

|  | *All patients in  hysterectomy analysis* | |
| --- | --- | --- |
|  | *Women  with VWD*  *(*n *= 1,374)* | *Women without VWD*  *(*n *= 12,791)* |
| Timing of VWD diagnosis / match date, *n* (%) | |  |
| Recorded in historical record | 726 (52.8) | 6,462 (50.5) |
| Recorded in active record | 648 (47.2) | 6,329 (49.5) |
| Year of first VWD diagnosis / match date, *n* (%) | |  |
| <1988 | 200 (14.6) | 1,799 (14.1) |
| 1988−1999 | 468 (34.1) | 4,316 (33.7) |
| 2000−2009 | 538 (39.2) | 5,056 (39.5) |
| 2010−2016 | 168 (12.2) | 1,620 (12.7) |
| Age at first VWD diagnosis / match date, *n* (%) | |  |
| <10 | 258 (18.8) | 2,272 (17.8) |
| 10−19 | 315 (22.9) | 3,017 (23.6) |
| 20−29 | 332 (224.2) | 3,244 (25.4) |
| 30−39 | 239 (18.2) | 2,226 (17.4) |
| 40−49 | 129 (9.4) | 1,194 (9.3) |
| ≥50 | 101 (7.4) | 838 (6.6) |
| Mean (SD), years | 24.7 (15.9) | 24.5 (15.2) |
| Median (range), years | 23 (0–85) | 23 (0–85) |

SD, standard deviation; VWD, von Willebrand disease.
